# Improving biology faculty diversity through a co-hiring policy and faculty agents of change

**DOI:** 10.1371/journal.pone.0285602

**Published:** 2023-05-15

**Authors:** Marissa Harris, Sue Rosser, Michael Goldman, Leticia Márquez-Magaña, Rori V. Rohlfs

**Affiliations:** 1 Department of Biology, San Francisco State University, San Francisco, California, United States of America; 2 Provost Emerita, San Francisco State University, San Francisco, California, United States of America; Georgia Southern University, UNITED STATES

## Abstract

Persons Excluded due to Ethnicity and Race (PEERs) remain underrepresented in university faculties, particularly in science, technology, engineering, math and medicine (STEMM) fields, despite increasing representation among students, and mounting evidence supporting the importance of PEER faculty in positively impacting both scientific and educational outcomes. In fact, the ratio of PEER faculty to students has been steadily dropping since 2000. In our case study, we examine the factors that explain creation of an unusually diverse faculty within a biology department. We analyzed nearly 40 years of hiring data in the study department and show that this department (the study department), historically and currently, maintains a significantly higher proportion of PEERs on faculty as compared to two national datasets. Additionally, we identify factors that contributed to hiring of PEERs into tenure and tenure-track positions. We observed a significant increase in the hiring of PEERs concurrent with the implementation of a co-hiring policy (*p* = 0.04) which allowed a single search to make two hires when at least one candidate was a PEER. In contrast, three key informants at sister departments reported that co-hiring policies did not result in PEER hires, but instead different practices were effective. In line with one of these practices, we observe a possible association between search committees with at least one PEER member and PEER hiring (p = 0.055). Further, the presence of particular faculty members (Agents of Change) on search committees is associated with PEER hiring. In this case study the combination of a co-hire policy based on the principle of interest-convergence to redress hiring inequities, along with the presence of agents of change, increased faculty PEER representation in STEMM departments.

## Introduction

Systemic barriers to engaging Persons Excluded due to Ethnicity and Race (PEERs) not only limit rigor, innovation, and relevance of scientific research, they perpetuate social inequities. With inclusion of PEERs comes diverse perspectives that improve scientific rigor by bringing more knowledge to bear on research questions, and by honing arguments in an inherently more critical setting [1]. Consequently, it is not surprising that papers co-authored by ethnically diverse groups are better cited [2]. Inclusion also promotes innovation [3], and an impactful focus on the health of communities of color [4–6]. This is because PEER scientists are more likely to pursue research topics that are relevant to communities of color than their non-PEER counterparts [7], despite topic-choice bias that often limits their own professional advancement [8]. Therefore, harnessing the potential of this exceptional commitment by increasing PEER representation in the scientific workforce may best address long-standing social inequities, for example, the excess burdens experienced by communities of color as a result of climate change [9] or the COVID-19 pandemic [10].

One systemic barrier to advancement of PEER students in the life sciences is the significant underrepresentation of faculty who share their social identities. While the “new majority” of science students often come from poor communities of color, are the first in their family to attend college, and are women [11], tenure-track and tenured science faculty continue to be primarily white and male [12]. It can be argued that these well-represented faculty, despite their best intentions, are less effective in advancing the “new majority” student for a variety of reasons. These may include unconscious bias in grading [13], which has not been well investigated in science settings, and the well documented need for authentic role models in classroom and research settings [14–17] to overcome psychosocial barriers to educational success like stereotype threat [18–21]. In fact, the outcomes of inequitable representation of student identities in the faculty may partially explain the inability of university programs and billions of federal dollars to significantly enhance the diversity of the scientific workforce [22, 23]. It appears that regardless of these investments, the disconnect between the overly non-PEER faculty and the students they are tasked to serve results in distrust of faculty by PEER college students [24].

Trust is a necessary component of effective faculty-student relationships (including teaching, training, advising, and mentoring) crucial to student advancement in science [25]. Trust has been linked to instructor immediacy, which is the apparent social closeness between a faculty member and student [26]. Instructor immediacy is associated with learning and academic success [27, 28], while ambient signals of non-inclusion can trigger underperformance of students as a result of stereotype threat [20, 21]. In fact, students from PEER groups in particular report a departure from science disciplines due to unwelcoming behaviors from science faculty [11] such as frequent microaggressions [29].

While inequity in faculty hiring harms “new majority” students, few successful approaches to attaining racial/ethnic faculty diversity have been published. Instead the focus has been on the publication of documented barriers, and guidelines for mitigating bias and promoting inclusive hiring [30, 31]. While these approaches have been met with modest success, still only white and some Asian populations are well-represented in academic science at the national level [12]. In academic medicine, PEERs are hired at significantly lesser proportions than their white counterparts [32]. It is clear that the pool of PEER candidates is substantial, but systemic racism and the need to protect Whiteness in higher education is pervasive [31, 33], requiring inventive solutions to hire and retain PEER faculty to best meet science student needs for success.

In the past, affirmative action policies were used to work towards compositional representation of students’ racial/ethnic identities among faculty, and to promote inclusive excellence. However, legal frameworks have changed. In California (the setting for this study), the passing of Proposition 209 in 1996 prohibited the use of affirmative action in hiring, and changes at the federal level further constrained diversity efforts [34–36]. Therefore, in California and elsewhere, institutions of higher learning experimented with a variety of options to encourage equity and diversity in faculty hiring to promote student success. For example, instead of using additional funds provided by the central administration to hire a single PEER as an “affirmative action hire,” pre-allocated funds would be used to hire a non-PEER, and additional funds from the central administration would be used to hire a PEER during the same search [34–36]. In this paper we use the term “co-hire” for this type of diversity effort and analyze the outcomes of faculty hiring during a 39-year period in a biology department (hereafter the study department) located in one predominantly undergraduate masters-granting institution on the West Coast. This analysis is further contextualized by factors related to best practices and challenges for recruitment and retention of PEER faculty.

To overcome documented barriers to recruitment and retention of PEER faculty [30, 37] it is important to consider the roots of U.S. higher education and the policies and practices that have led to its evolution. Higher education in the newly founded U.S. was used as a tool for creating a monocultural and monolingual society, and only recently have pedagogical approaches emanating from critical race theory that embrace cultural pluralism and equality been described as critical to the educational success of minoritized students [38]. These pedagogical approaches were first advanced in the mid-1990s at the same time that affirmative action was being dismantled in California. Nonetheless, at this time, and for decades later, the biology department that is the site of this investigation (i.e., the study department) successfully recruited and retained PEER faculty into its department of biology. We hypothesized that this study department has an unusually diverse faculty, both historically and currently, and maintains a significantly higher proportion of PEERs on faculty as compared to faculty at other undergraduate, masters-granting institutions nationally. We therefore conducted retrospective data analyses to examine the composition of faculty in the study department and compared that with national data at similar universities. We examined factors implicated in inclusive hiring [31] including 1) the impact of an informal co-hiring policy, 2) if the presence of a PEER on search committees increased the chance of making at least one PEER hire as well as retaining PEER faculty, and 3) the role of faculty Agents of Change (AoC) (especially on search committees) in improvements in hiring diversity observed within the study department. These factors were contextualized by national trends in faculty hires and hiring practices in three departments at similar master’s-granting institutions.

## Methods

### Dynamic definition of PEER

In this study we use the acronym PEER, which was coined as the acronym for Persons Excluded because of their Ethnicity or Race [39]. At the same time, we recognize as biologists that race is a social construct, which is intrinsically dynamic because of its sociopolitical nature grounded in eugenics [40, 41]. The power of this social construct is clear in the impact of racism in academia. We aim to address some outcomes of this racism, and for the purposes of this study, follow the recommendation that racial/ethnic categories be used to measure the success or failure of particular policies, despite the inherent flaws of these categories [42]. For example, demographic composition can vary between settings and will therefore change the distribution of representation [43]. Additionally, the dynamic, and sociopolitical nature of race and ethnicity means that any definition of PEER runs the risks of excluding racial/ethnic groups that are underrepresented (e.g., Southeast Asian) [44, 45], and including groups who are actually overrepresented and/or not historically excluded (e.g., White individuals who claim Native American ancestry) [46]. Here, for our quantitative analysis we relied on currently accepted criteria (noting concerns in the Discussion section) and have used the National Institute of Health (NIH) definitions. The NIH defines underrepresented minority groups as racial and ethnic groups that have been shown to be historically underrepresented in biomedical research: individuals who are Black or African American, Latinx, American Indian or Alaska Native, Native Hawaiian and other Pacific Islanders [43].

As a comparison, we performed a parallel analysis based on gender and offer a new term. Using the same principles used in naming PEERs, we propose Persons Excluded because of their Gender (PEGs), which includes, for example, scientists who are two-spirit, transgender, genderqueer, gender fluid, non-binary, gender non-conforming, and cisgender women. Because of the expansive and dynamic nature of gender, it may be more precise and long-lasting to define PEG by its complement non-PEG, which includes cisgender men.

### Data sources

For this analysis we drew data from national databases, the study department, and departments at sister institutions. Table 1 summarizes these data sources and the research questions which we address with them.

**Table 1:** Data sources and research questions.

Data source	Questions addressed
National data^+^ WMPwD (average n = 70,227 faculty per year) IPEDS (average n = 512 institutions per year)	Does the study department have an unusually high proportion of PEER faculty?
Study department data* Departmental faculty (n = 94 faculty) Hiring data (n = 69 hires) Search committee membership (n = 48 searches, n = 68 search committee members)	In the study department and other institutional contexts, how is PEER hiring impacted by the co-hiring policy, by the presence of PEERs on search committees, by the actions of AoCs on search committees and in junior faculty support?
Sister department data Key Informant interviews (n = 3 informants)	

^+^ Annual WMPwD and IPEDS *n* values are in S1 Table

* Note that search committee membership data were not available for older historical hires

IPEDS = National Center for Education Statistics (NCES) Integrated Postsecondary Education Data System

WMPwD = National Center for Science and Engineering Statistics (NCSES) report entitled, “Women, Minorities, and Persons with Disabilities in Science and Engineering”

AoC = Agent of Change

#### Study department data

We calculated the total faculty employed in the study department across 74 years (n = 94). Of these, we were able to compile more detailed data (including hire date, search committee composition, PEER status, and PEG status) on tenure or tenure-track faculty hires (n = 69) for a period of 39 years. These data can be divided into three time periods delineated based on when co-hiring policies (CHP) were active (pre-CHP, active CHP, and post-CHP).

To enumerate all faculty hires in the study period, we collected surveys at two biology department meetings in 2015 and conducted follow-on informational interviews with five long-term and emeritus departmental faculty. We used university bulletins to verify when faculty were hired and the time period of their hire. When bulletin data was inconsistent with historical knowledge of department members (such as some cases where faculty known by the authors to still be present in the department were not listed in the bulletin), we cross-referenced the bulletin data with archived faculty rosters, the current department website, and additional informational interviews with faculty who had left the department. End dates of employment were determined from departmental data and confirmed (where possible) by university bulletins.

Our data include socially assigned PEER and PEG statuses for each hire and for the members of search committees [47]. Per the institutional review board (IRB) approved protocol for this case study, current faculty were contacted for informed consent. Retired faculty were exempt and not contacted due to minimal risks of loss of privacy and the fact that many could not be contacted (e.g. deceased, no contact information). Consequently, self-identification was not possible and socially-assigned demographics were used [47]. While using socially assigned identities is not ideal and is a limitation to our analysis, it provides socially-relevant information on the historical dataset used in this case study.

For the most recent 31 years of data, we also assessed which faculty members served on a search committee (n = 68) and determined the composition of each search committee (48 searches) using: 1) historical records from previous department chairs; 2) self-report of service on a search committee; and 3) reports by faculty hires of who served on their committee.

#### National data

We compared the percent of PEER faculty in the study department to the percentage of PEER faculty nationwide. We used publicly available data compiled by 1) the National Center for Education Statistics (NCES); and 2) the National Center for Science and Engineering Statistics (NCSES). We considered the NCES Integrated Postsecondary Education Data System (IPEDS) (referred to as the IPEDS data set) [48]; and the National Science Foundation/NCSES biennial report entitled, “Women, Minorities, and Persons with Disabilities in Science and Engineering” (referred to as the WMPwD data set) [49].

The ideal data to compare the study department to the national faculty population would be biology departments at predominantly undergraduate, masters-granting institutions. However, this resolution of comparison data was not available. Instead we obtained two populations to analyze: faculty employed at masters-granting institutions across disciplines (IPEDS); and faculty within biological life sciences across institutional settings (WMPwD).

The IPEDS data set compiles data from every college, university, and technical/vocational institution that participates in the federal student financial aid programs. Using this database, we searched for full-time tenured and tenure-track instructional faculty by race/ethnicity and gender employed at institutions with the same masters-granting classification as the university housing the study department. Furthermore, we only used data from years when data submission was mandatory [50]. We describe these, and other, caveats of the available data for each year in S1 Table.

The NSF/NCSES report, WMPwD, covers a wide range of topics encompassing the education and employment of PEERs and PEGs in science and engineering. Within the Employment section, we focused on data tables that showed race/ethnicity and gender of Science & Engineering doctorate holders in the biological sciences employed at postsecondary institutions. Each report publication year had slightly varying data available regarding the tenure status and disciplines of the faculty surveyed (see S1 Table).

In summary, the IPEDS data included faculty at nationwide masters-granting institutions and included all departments, not just biological sciences. The WMPwD data included nationwide faculty explicitly in biology, but combined data across undergraduate, masters-granting, and PhD-granting institutions. For our analysis, these datasets are complementary. The IPEDS data allowed us to compare data about the study department to faculty explicitly at masters-granting institutions across departments, while the WMPwD data allowed for comparison to faculty explicitly in biology across many institution types.

Another important difference between these data sets is their racial/ethnic categorization schemes. In the IPEDS data system, individuals who are not U.S. citizens, nor permanent residents are classified as "nonresident aliens" and are disaggregated from the other race/ethnicity groups. IPEDS does not have race/ethnicity data for the “nonresident aliens” group so these respondents are included in our total faculty count but are not included in our PEER designation. In the WMPwD data set, all scientists and engineers who reside in the U.S. are included in the race/ethnicity data regardless of citizenship or immigration status. Therefore, our WMPwD PEER designation includes all respondent data regardless of citizenship. To accommodate these varying criteria for PEER, we performed our analyses with the WMPwD PEER criteria in the main text and with the IPEDS PEER criteria in the supplement.

Race/ethnicity categorization also varies across the longitudinal data available for IPEDS and WMPwD. In some cases, respondent data for a particular racial/ethnic category was suppressed because the case count was too small and therefore at risk of breaching confidentiality (see S1 Table for details); thus, prohibiting this respondent data from being counted in our PEER sample. There were also changes in race/ethnicity categories over time. For both data sets, earlier years had fewer, broader categories (i.e., “Asian/Pacific Islander”). Starting around 2005, both data sets began to disaggregate their data into more distinct categories (i.e., “Asian” and “Native Hawaiian/Other Pacific Islander” as two separate categories). This affected the PEER analysis in that the “Asian/Pacific Islander” respondent data from earlier years was designated as non-PEER, per the NIH guidelines, even though it included a PEER group (“Pacific Islander”).

The treatment of multiracial individuals is also lacking. Both national data sets disaggregated multiracial individuals into a separate cumulative category, which impeded assessment of multiracial identities as PEER or non-PEER. In fact, there is a scarcity of research on the representation of multiracial identities in academia [51]. With these limitations, respondent data from the individuals in the “more than one race” category were included in the overall totals in this study but were not designated as PEER. Additionally, the NIH criteria of racial/ethnic groups are limited in their assessment of distinct nationalities. For example, the definition for “Asian” includes persons with ancestry from Asia, including, but not limited to, ten different countries [52]. Categorizing multiple nationalities into one racial/ethnic category eliminates the capacity to analyze representation within the category itself.

The binary scheme to categorize individuals as PEER or non-PEER is limited as it does not attempt to describe complex racial or ethnic identities, nor relevant intersectional identities. This analysis is limited to non-intersectional data as the study department is too small to disaggregate into a more sophisticated model that accounts for intersectional identities. Moreover, doing so would leave our analyses statistically underpowered, and could increase the risk of individual identifiability.

Similar to the limitations posed by a binary PEER/non-PEER schema, the national data regarding gender is denoted into only two gender categories: “male” and “female.” This cisnormative gender categorization scheme does not take into account most PEG identities. While recognizing the limitations of this binary gender schema, due to lack of more accurate data, respondent data from the “female” category was classified as PEG and respondent data from the “male” category was classified as non-PEG. Working across these distinct classification schemes poses a limitation to our analysis comparing the study department to national data.

Despite these limitations, the PEER/non-PEER and PEG/non-PEG categorization schemes do allow us to investigate broad scale trends in equity in faculty hiring. Therefore, while acknowledging the limitations, we move forward with our analysis.

### Sister institutions

To gain broader context about the impact of co-hiring, PEERs on search committees, and AoCs in different institutional contexts, in-depth interviews were conducted with leaders (n = 3) in two Biology departments and one Chemistry & Biochemistry department from similar institutions. These three professors were hired at three other master’s granting institutions over 30 years ago and were able to provide a historical account of faculty hiring since the ban on affirmative action in 1996. They served as a convenience sample for the interviews because they were known as departmental leaders to the co-author that interviewed them. Moreover, all three reported that their departments had utilized a co-hire policy for 2–3 years following the ban on affirmative action making them additionally suitable for inclusion in this case study. To learn about the nature and outcomes of these policies one co-author conducted open-ended interviews with questions informed by our analyses of hiring data compiled from the study department. The questions focused on the existence and importance of co-hire practices at sister institutions, the general nature of the search committees that led to successful hires of PEER candidates and other departmental practices that may have played a role. Notes collected from the interviews were shared with 3 of the co-authors and findings discussed and organized into themes in accordance with grounded theory approaches to qualitative research [53, 54]. The answers obtained are limited by the small number of individuals who were interviewed, the accuracy of key informant memory, and both their conscious and unconscious bias.

### Ethics statement

The Institutional Review Board at the study department university reviewed and approved this study (protocol #H19-13). Verbal informed consent by current faculty was obtained for participation.

### Analysis

#### Quantifying study department hiring longitudinally

We computed the rate of study department PEER hiring over three time periods based on when the co-hiring policy was active: a period of 12 years before the start of the co-hiring policy (pre-CHP), a period of 17 years when the co-hiring policy was active (CHP), and a period of 10 years after the end of the co-hiring policy (post-CHP). For context, affirmative action policies were in place during pre-CHP and the first year of CHP, but were repealed the second year of CHP and remained banned for the rest of CHP and post-CHP. We tested for increases in the rate of hiring for PEERs and PEGs between the pre-CHP to the CHP using a one-tailed Fisher exact test. We used a two-tailed Fisher exact test to examine whether the hiring rates shifted between the CHP to the post-CHP.

#### Testing search committee composition and PEER hiring

We tested for correlation between a search committee having at least one member with a PEER or PEG identity, versus that committee hiring at least one individual with a PEER or PEG identity. For example, we made a contingency table for search committee composition (containing at least one PEER member or not) and identity of hire(s) (at least one PEER hire or not) (S2 Table). For each of these contingency tables, we performed a one-tailed Fisher exact test and computed the odds ratio. Note that the first PEER faculty hire was necessarily done by an all-non-PEER committee. To accommodate this constraint, we performed a modified Fisher exact test to accounting for this specific contingency limitation. Because the earliest hiring data available to us lacked search committee data, we performed this analysis on a set of 48 searches performed over 31 years by a cumulative 68 search committee members.

#### Identification of Agents of Change

We investigated if particular individuals’ search committee service is associated with PEER hiring success as evidence of the presence of “Agents of Change” (AoCs). To determine AoC status, for each individual who served on a search committee (*n* = 68), over 48 searches, we considered the outcome (at least one PEER hire or not) of searches where they were on the committee, versus the searches where they were not on the committee. We performed one-tailed Fisher exact tests on the resulting contingency tables.

Similarly, we considered if, of the 18 total PEER hires, PEER hires with at least one PEER on their search committee are more likely to be retained than their PEER hire counterparts without any PEERs on their search committees.

We went on to determine if there is cumulative evidence that search committees including particular individuals are more likely to result in a PEER hire. Specifically, we investigated if the observed number of associations between individual search committee members and PEER hiring success is more than would be expected if there were no underlying relationship between search committee membership and PEER hiring success. We performed a permutation test, shuffling the hiring outcomes versus search committees and testing for the same associations. We performed 10,000 such permutations. We report on the proportion of permutations that had at least as many individuals with Fisher exact test *p*-values below 0.1, as compared to the empirical data. We use this metric of *p*<0.1 because the per-search-committee-member sample size of searches is low, with 43 of the 68 committee members sitting on just one or two search committees. So, the lowest possible *p*-value for those committee members who sat on two committees that each made a PEER hire is 0.08. Using a higher p-value threshold allows us to consider these data with small sample sizes [55].

#### Key information from sister institutions

In-depth interviews (n = 3) scheduled for one-hour were conducted to understand the factors that impacted hiring of PEER faculty in STEMM departments at three similar institutions. These interviews help us understand the potential role of co-hiring, PEERs on search committees, and AoCs in different settings. The following interview questions were asked at each meeting: How long have you been a member of your department? Who was on your search committee in terms of men/women, faculty of color? Do you/your department maintain historical hiring data, and can you tell me the racial/ethnic demographics of your current faculty? Have you ever had a co-hire policy in place? If so, what were the outcomes? Have you served on a search/screen committee (as possibly the only PEG or PEER), and what was the outcome/your experience? Does your department use a codified rubric/criteria to select candidates for on-campus interview? These questions had been developed based on the analysis of what appeared to have worked at the study department and focused on possible Agents of Change, search committee composition, co-hire policies, and strategies to reduce bias in selection.

#### Comparison to national data

We used a chi-squared test to identify years when the study department had a significantly higher proportion of PEER faculty as compared to the WMPwD data set. We calculated the empirical *p*-value to identify years when the study department had significantly higher proportions of PEER and PEG faculty than in the IPEDS data set.

## Results

### Relatively high faculty PEER proportion in study department

**Fig 1:**
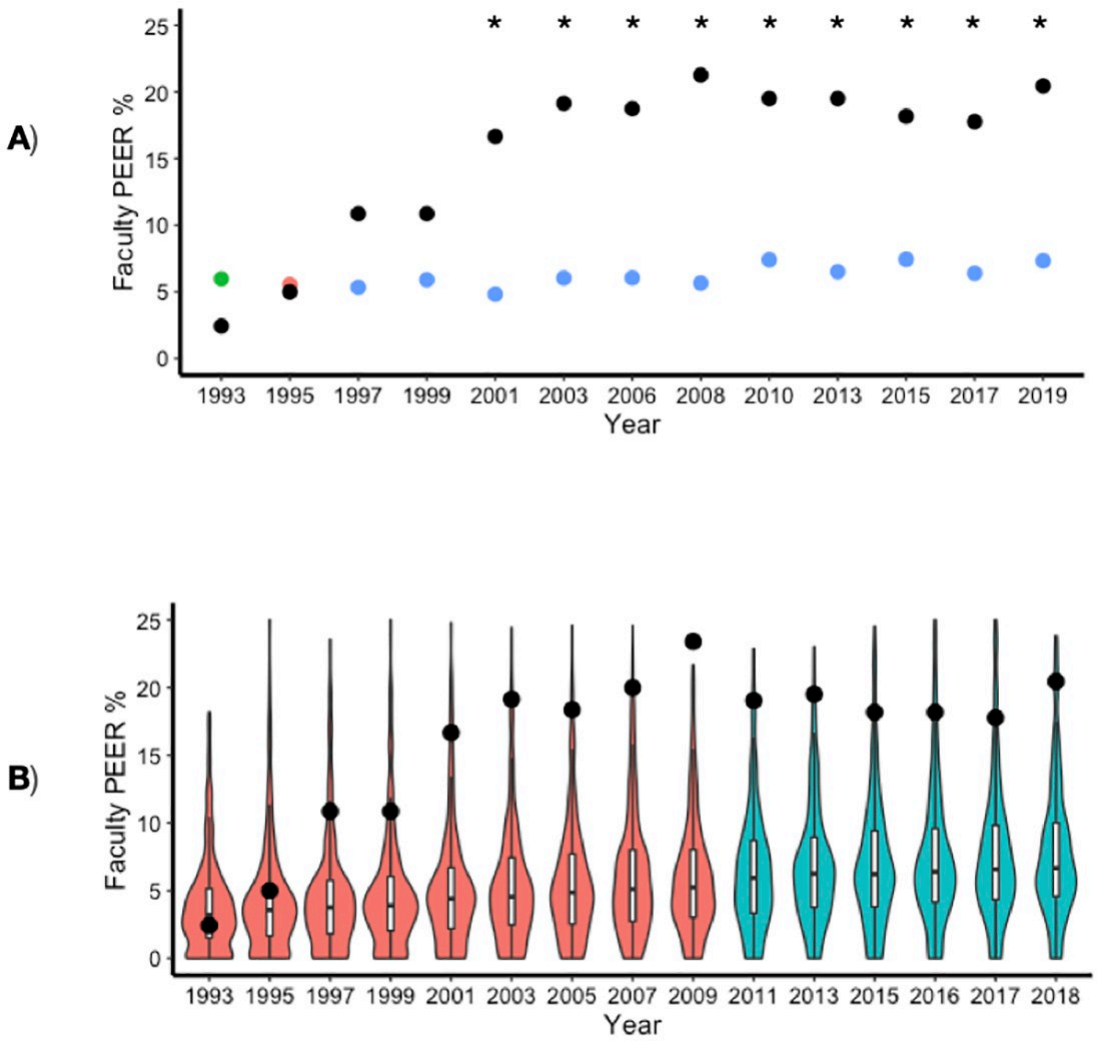
PEER demographics in study department compared to national science & engineering faculty. A) The percentages of PEER faculty are shown for both the study department (n = 94 faculty) and nationwide science & engineering doctorate holders (average n = 70,227 faculty per year) as listed in the WMPwD report for the years that WMPwD was published. The study department percentages are indicated by black points and the nationwide science & engineering doctorate holders are indicated by green, red, and blue circles. For the nationwide data, colors represent the survey sample specification–science & engineering doctorate holders employed in all postsecondary institutions (green); science & engineering doctorate holders employed at 4-year colleges and universities (red); and tenured and tenure-track doctorate holders in the biological sciences employed at 4-year colleges and universities (blue). Years with significantly higher PEER percentages in the study department faculty compared to nationwide faculty are indicated by stars. B) The percentage of PEER faculty at the study department (black points) and nationwide faculty at 4-year institutions (violin plots) as reported in IPEDS for the years with mandatory reporting (average n = 512 institutions per year). The orange violin plots represent the years where Asian and Pacific Islander ethnicities were classified in one group considered non-PEER. The teal violin plots represent the years where there were two separate categories: 1) Asian, and 2) Native Hawaiian or Other Pacific Islander. For those years, the Native Hawaiian or Other Pacific Islander category was included in the PEER percentage.

The proportion of PEER faculty in the study department increased over the study period, a trend that was not reflected in the national life sciences faculty data. After the initial PEER hire, the proportion of PEER faculty in the study department increased and surpassed the national average of biology faculty across institutional settings by approximately 6% (Fig 1a). The study department held a significantly higher percentage of PEER faculty than the WMPwD comparison population starting in 2001. We additionally used the IPEDS data to compare the percentages of PEER faculty in the study department to tenured and tenure-track faculty at comparable masters-granting universities nationally (Fig 1b). After 2003, the years of available data differ between the IPEDS data set and the WMPwD data set because each followed a different schedule of data collection. Starting in 1997, we observed higher percentages of PEER faculty in the study department compared to the median percentage of PEER faculty across departments in similar institutions nationwide. It is noteworthy that the study department had a higher percentage of PEER faculty than some entire faculties (including STEMM and non-STEMM departments) during these years.

As a comparison, we compared the percentage of PEG faculty within the study department to the percentage of faculty categorized as “female” within the two national faculty data sets. While these classifications are not identical, we know of no historical national-scale data with more accurate historical gender information. The percentage of PEG faculty in the study department experienced a 17% increase between 1999 and 2015 (S1 Fig). In 2015 and 2017, the study department had a significantly higher proportion of PEG faculty as compared to WMPwD data from science & engineering departments across institutional settings (S1A Fig).

However, compared to the IPEDS data with STEMM and non-STEMM faculty at comparable master’s-granting institutions (average n = 513 institutions per year) (S1B Fig), the percentage of PEG faculty in the study department is consistently low for all years observed, except 2015–2016 where it meets the nationwide average percentage of PEG faculty. This is likely due to the fact that the comparison sample includes faculty from all disciplines at master’s granting institutions that have higher percentage of women faculty [56]. For some years, institutions may have reported faculty gender demographic information but not faculty racial/ethnic demographic information, hence the average sample size of institutions per year in the IPEDS dataset differs between the two comparisons. The specific sample sizes for each comparison are listed in S1 Table.

#### Worsening national PEER faculty to student ratios

**Fig 2:**
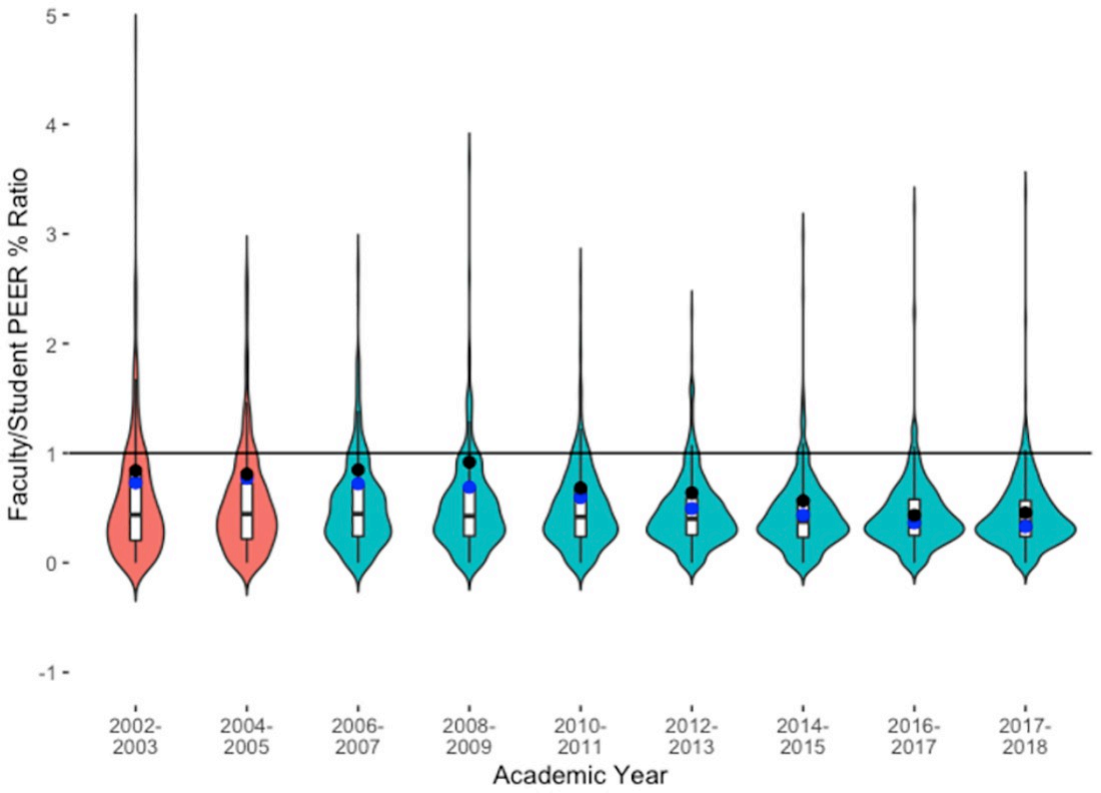
Ratio of PEER faculty to students in study department compared to national master’s-granting institutions. This plot shows the ratio of PEER faculty percentage/PEER student percentage at the study department (black points), at the study institution (blue points), and the ratios of PEER faculty percentage/PEER student percentage at institutions nationwide (n = 673 institutions per year) as reported in IPEDS (violin plots). The horizontal line represents the ratio of one, where the faculty PEER percentage and student PEER percentage would be equal. Ratios above one indicate institutions with higher PEER faculty percentages than PEER student percentages. Ratios below one indicate institutions with lower PEER faculty percentages than PEER student percentages. The colors of the violin plots reflect the differences in IPEDS race/ethnicity categorizations: the orange violin plots represent the years where Asian and Pacific Islander ethnicities were classified together and the Pacific Islander ethnicity was not included in the PEER percentage, while the teal violin plots represent the years where there were two separate categories: Asian; and Native Hawaiian or Other Pacific Islander; and the Native Hawaiian or Other Pacific Islander category was included in the PEER percentage. For the study department, “Native Hawaiian or Other Pacific Islander” was included in the PEER percentage for all years.

While the study department’s increase in faculty PEER representation is a welcome change, it occurred in a time when student PEER representation was also improving. As a preliminary investigation into changes in these PEER students’ access to PEER faculty, we consider the ratio of the percentage of PEER faculty versus the percentage of PEER students. We examine this ratio longitudinally for the study department and for the national IPEDS data across departments in masters-granting universities (Fig 2). A ratio of one indicates the faculty PEER percentage in an institution or department is equal to the student PEER percentage in the same institution or department. Ratios above one indicates higher PEER faculty percentages than PEER student percentages, and ratios below one indicates lower PEER faculty percentages than PEER student percentages. The proportion of institutions in our national dataset with a lower proportion of PEER faculty compared to students (ratio < 1) increased from 84.1% in 2003 to 95.9% in 2018 (S3 Table). While the ratio of PEER faculty to students in the nationwide data does not change significantly from one year to the subsequent available year (S4 Table), there is a significant decrease when comparing academic years 2002–2003 to 2017–2018 (t-test, p<0.001). This worsening of PEER faculty representation compared to students reflects how modest advances in PEER faculty hiring are still insufficient in the face of student demographics.

The study department exhibited a slightly higher PEER faculty to student ratio than the national average ratio from academic years 2002–2003 to 2014–2015, reflecting a proportion of PEER faculty at the study institution that is closer to the percentage of PEER students, as compared to comparable institutions (S3 Table). However, the increase in PEER students outpaced PEER faculty representation, as the ratio dropped over time.

### Factors implicated in inclusive hiring

Our national analysis shows that the study department is exceptional in its PEER faculty representation. We now investigate factors that may have supported this shift, specifically the co-hiring policy, the presence of PEERs on search committees, and the actions of AoCs.

#### Faculty PEER representation improves with co-hiring policy

**Fig 3:**
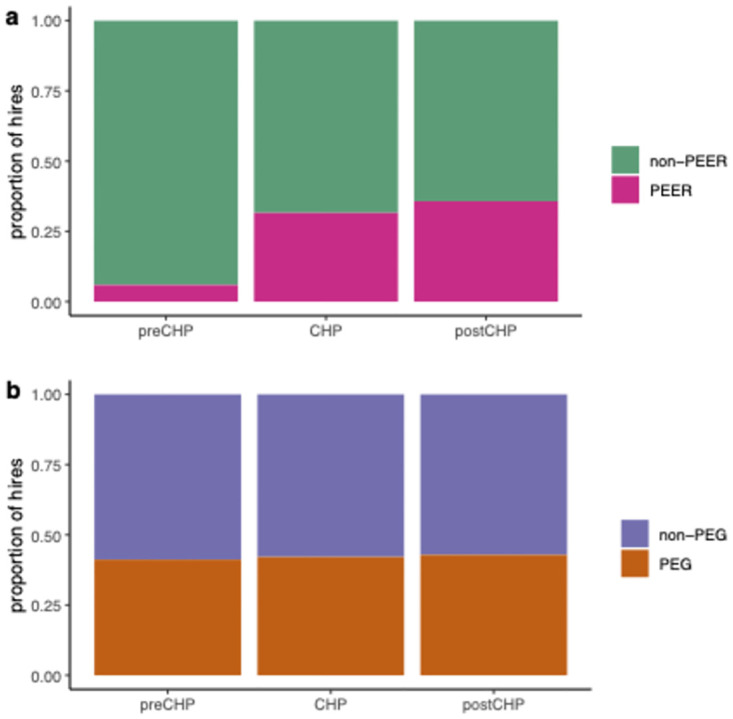
Departmental hiring of before, during, and after CHP. These plots show changes in proportion of faculty hired (n = 69) before the CHP, during the CHP, and after the CHP in terms of (a) faculty hires who are non-PEERs (green) and PEERs (pink), and (b) faculty hires who are non-PEGs (purple) and PEGs (orange).

In examining factors that may support inclusive hiring practices, we considered the impact of the co-hiring policy by comparing the rate of PEER hires before (pre-CHP), during (CHP), and after (post-CHP) (Fig 3, Table 2). We contrasted this to changes in the rate of PEG hires, which are not expected to be impacted by the co-hiring policy. Due to the complex dynamics of finances, enrollment, departmental needs, and other factors, the total rate of hiring varied between pre-CHP (17 hires), CHP (38 hires), and post-CHP (14 hires). The rate of PEER hiring increased significantly from 5.9% during the pre-CHP to 31.6% in the CHP (*p* = 0.04) and was maintained into the post-CHP at 35.7% (*p* = 1.0). By contrast, the rate of PEG faculty hires, which were not targeted by the co-hiring policy, remained constant from 41.2% in the pre-CHP to 42.1% in the CHP (*p* = 0.59), to 42.9% in the post-CHP (*p* = 1.0).

**Table 2:** Demographic composition of hires before, during, and after CHP.

	Pre-co-hiring period	Co-hiring period	Post-co-hiring period
**Non-PEER hires**	16 (94%)	26 (68%)	9 (64%)
**PEER hires**	1 (6%)	12 (32%)	5 (36%)
**Non-PEG hires**	10 (59%)	22 (58%)	8 (57%)
**PEG hires**	7 (41%)	16 (42%)	6 (43%)
**Total hires**	17	38	14

#### Search committee composition and Agents of Change

In addition to the impact of co-hiring policy and other informal policies on hiring, we investigated if the presence of a PEER on search committees impacted the chance of making at least one PEER hire. We performed parallel analyses considering gender. Specifically, we investigated whether 1) committees with at least one PEER were more likely to make at least one PEER hire, 2) committees with at least one PEG were more likely to make at least one PEER hire, 3) committees with at least one PEER were more likely to make at least one PEG hire, and 4) committees with at least one PEG were more likely to hire at least one PEG (S2 Table, Table 3). Our results show that search committees with at least one PEER member were 3.4 times more likely to have had one or more PEER hire (*p* = 0.055; odds ratio of 3.4; Table 3). We observe a similar, but weaker, relationship between the search committees with at least one PEER member and successful PEG hiring (*p* = 0.073; odds ratio of 2.9; Table 3). We do not observe evidence that committees with at least one PEG are more successful in hiring either PEER or PEG faculty.

**Table 3:** Correlation between search committee composition and hiring demographics.

Search Committee Composition	Fisher exact *p*-value	Odds ratio
>= 1 PEER on committee versus >= 1 PEER hire	0.055#	3.4
>= 1 PEG on committee versus >= 1 PEER hire	0.305	2.2
>= 1 PEER on committee versus >= 1 PEG hire	0.073	2.9
>= 1 PEG on committee versus >= 1 PEG hire	0.763	0.79

^#^: adjusted Fisher exact test to account for the contingency limitation of the first PEER faculty member hired by entirely non-PEER committee

Knowing that search committee composition could have an impact on PEER hiring success, we went on to investigate if there were particular individuals whose presence on a search committee is associated with this success. We found evidence of this association and refer to these individuals as Agents of Change (AoCs). Of the 68 search committee members analyzed, one individual was significantly associated with PEER hiring success (*p* = 0.021, one-tailed Fisher exact test), and eight others showed weaker associations with *p*-values below 0.1 (S2 Fig).

To determine if this reflects high individual-level association with PEER hiring success, we performed a permutation test, calculating the same *p*-values while shuffling search outcomes. We found that only 0.0329 of permutations resulted in at least nine individuals with *p* < 0.1. This suggests that indeed the historical data reflect a subset of individuals having a disproportionate impact on PEER hiring success.

Additional patterns emerge when considering the composition of search committees and the retention of PEER hires. Over all PEER hires, we observe non-independence between having a PEER on the search committee, and retention through tenure (p = 0.052, two-tailed Fisher exact test). Of the 18 total PEER hires during the study period, 14 were retained and 4 left the study department.

### PEER faculty representation and hiring policies across departments

Through our interviews with three key informants from sister institutions, we learned department demographics and implementation of institutional support for three comparable departments (Table 4). While these do not constitute formal controls and may be impacted by respondent’s recall ability over their career span, they provide context for the varying impact of similar policies in different environments. For each of the sister departments and the study department, the table shows the PEER and PEG faculty percentages at the time of key informant interviews, which took place during the last 5 years. Two of these individuals, Key Informants (KIs) 1 and 2, are PEERs who participated in the diversification of their respective departments; in concordance with their efforts, the percentage of PEER faculty was 16% and 38%, respectively, at the time of their interviews. The third individual (KI3) worked to increase the proportion of women in her department but reported a lack of PEER hires resulting in 0% PEER faculty in her department. At the time of the interviews, the study department had 20% PEER faculty, the second highest of the four departments.

**Table 4:** Sister institution and study department demographics.

Departments	% PEER	% PEG
**KI1**	**16%**	**34%**
**KI2**	**38%**	**44%**
**KI3**	**0%**	**45%**
**Study Dept**	**20%**	**43%**

While we observed a significant increase of PEER hiring in the study department during the implementation of co-hiring, this was not found at sister institutions. The brief co-hiring periods (2–3 years) at sister departments did not impact PEER hiring. Instead, key informants discussed informal policies (described below) as being critical contributors to increased hiring and retention of PEER faculty in two of the three sister departments.

KI1 reported the effective recruitment of PEER candidates through personal connections and strategic networking with science organizations having high PEER representation. She also reported the retention of PEER hires via an informal practice of assigning the chair of the successful search committee as the mentor for the incoming PEER faculty member. KI2 reported being recruited to an open faculty position as a result of the department’s informal policy to recruit its own alumni with family ties in the local community. He also reported the success of this informal policy in recruiting other PEERs.

KI3 reported the lack of success in her department to draw PEER candidates to open positions despite good intentions. These included maximizing diversity on search committees through inclusion of women and individuals from well-represented non-white ethnic groups, and a requirement for a diversity/student success statement as part of the application materials.

The influence of an AoC is observed in the departments of our key informants as well. KI1 and KI2 reported that successful searches for additional PEERs were supported by early PEER hires, as well as by the use of informal policies. In particular, after KI1 earned tenure, she served on seven search committees which led to the hiring of women and faculty of color. KI2 reported that the first PEER hire in his department participated on search committees that hired other PEER faculty, including the search for his own position.

## Discussion

Our results show that the study department has an exceptional density of PEER faculty. Our attempt to understand the causes of this representation provides a case study illustrating the collective impact of policy and AoCs to enhance racial/ethnic diversity of science faculty. We expect this to be helpful for institutions that accept the call to action issued by thousands of PEERs in science to combat systemic racism in higher education [33].

First, we find that the proportion of PEER hires in the study department increased significantly during the co-hire period (CHP) and exceeded the proportion of PEER hires in two national datasets for the same time period. In contrast, key informants at sister departments reported disuse of informal co-hire policies.

Next, we observe that the successful hiring of PEERs was associated with the search committee including at least one PEER member. PEER faculty hired with PEERs on their committee were also more likely to be retained, perhaps because of ongoing mentorship from that committee member. We also see that certain AoCs contributed to successful PEER hiring in the study department. Similarly, key informants at the two sister departments with high rates of PEER hiring reported the success of informal strategies, faculty AoCs, working through faculty governance systems, and ongoing professional support of new PEER hires.

In examining the collective impact of the co-hire policy and AoCs, we consider critical race theory. The tenets of critical race theory have been utilized as a framework to analyze the efforts of diversifying higher education in previous scholarship [57–59]. Within this theory, the interest-convergence dilemma argues that progress in racial equity is made when the interests of whites and non-whites converge, but when interests diverge, progress is halted or lost [60]. It can be argued that the informal co-hire policy investigated in this report aligned the interests both of faculty/administrators working to improve racial/ethnic diversity, as well as of those less concerned with racial/ethnic representation but motivated to hire more faculty overall and/or to increase funds for research.

Our finding that PEER faculty were more often retained when a more senior PEER was on their search committee is consistent with the hypothesis that when a PEER faculty is on a search committee, that person is likely to support the new PEER hire through their junior years. It is also in alignment with the work of others that show that retention of PEER faculty is improved by culturally-congruent mentorship [61], which can partially mitigate the racial toxicity of higher education environments [37]. This pervasive and substantial toxicity varies across institutions, but it is often due to interest divergence [62] where the interests of the dominant group are prioritized. This possibility is consistent with the fact that after the end of the CHP, hiring priorities appeared to shift as described below.

While the end of the CHP brought a slight increase to the rate of hiring PEER faculty according to our WMPwD PEER criteria (includes all scientists regardless of citizenship or resident status), we see a non-significant decrease based on our IPEDS PEER criteria (includes only U.S. citizens and permanent residents). (S3 Fig, S5 Table). Following the end of the CHP that supported a specific type of PEER diversity, this shift aligns with apparent increased interest in different diversity factors including spousal hires and membership in other underrepresented groups in science. While we celebrate gains in other axes of diversity, we pause to consider that the departmental criteria for ‘diversity’ should be intentionally set based on the specific inequity that is being addressed. For instance, if the concern is increasing visible role models and opportunities for students at an institution with a predominantly domestic PEER population, then it may be warranted to prioritize hiring U.S.-trained PEER faculty [32]. This is in alignment with recommendations for increasing PEER representation and career opportunities in STEMM environments to make science more inclusive [32, 63].

Overall, our results and the varying dynamic criteria for PEERs support the need to use specific and intersectional approaches for understanding and addressing systemic inequality in faculty hiring. In 2020, federal agencies outlined intersectional approaches in the design of systemic change strategies that recognize that race and ethnicity, gender, and sexual orientation do not exist in isolation from each other and from other categories of social identity [64]. While we consider data from the study department a rich source of information, the sample size was too small to adequately protect individual privacy if using an intersectional approach. Therefore, to mitigate the risk of loss of privacy we report only aggregated findings and refrain from intersectional analyses.

We acknowledge there are confounding factors not included in our analysis that may affect success of using a co-hiring policy change and AoC for improved racial/ethnic diversity in faculty hiring (e.g., geographic location, institutional budgets, and departmental climate). Nonetheless, our results suggest that one possible step to sustainable PEER hires is formal and specifically targeted co-hire policies. At the same time, we note that a limitation of such a policy is the decades of implementation that would be necessary to remediate historical inequities in faculty hiring [12, 32]. Therefore, we recommend systemic incentives to spur interest-convergence [60] (for example, explicitly linking state and federal funding to gains in PEER hiring). Additionally, we recommend supporting and incentivizing AoCs to act in the faculty hiring process. Finally, for all diversity hiring policies we recommend that intersectionality, domestic vs. international training, and efforts to retain PEERs beyond hiring be carefully considered. After all, the “new majority” students in science have intersectional identities that must be better reflected in the faculty to build trust and success [24] and this requires not only recruitment of individuals like themselves, but their retention as valued faculty members.

## Supporting information

S1 Fig PEG demographics in study department compared to national science & engineering faculty. A) The percentages of faculty that are PEGs are shown for both the study department (n = 94 faculty) (black) and nationwide science & engineering doctorate holders (average n = 70,277 faculty per year) (green, red, and blue circles) over time. For the nationwide data, colors represent the survey sample specification–science & engineering doctorate holders employed in all postsecondary institutions (green); science & engineering doctorate holders employed at 4-year colleges and universities (red); and tenured and tenure-track doctorate holders in the biological sciences employed at 4-year colleges and universities (blue). Years with significantly higher PEG percentages in the study department faculty compared to nationwide faculty are indicated by stars. B) The percentage of PEG faculty at the study department (black points) and nationwide faculty at 4-year institutions (violin plots) as reported in IPEDS (average n = 513 institutions per year).
(TIF)

S2 Fig Evidence for presence of Agents of Change acting on search committees. Histogram of AoC -log(*p*-values). This histogram shows -log(*p*) from one-tailed Fisher exact tests querying individual search committee membership (n = 68 search committee members) association with hiring at least one PEER (IPEDS criteria) for the empirical historical data (orange) and for the permuted data (10,000 permutations) (purple).
(TIF)

S3 Fig Departmental hiring of before, during, and after CHP, using IPEDS criteria for PEER. These plots show changes in proportion of faculty hired (n = 69) before the CHP, during the CHP, and after the CHP in terms of a) faculty hires who are non-PEER (green) and PEER (pink) and b) faculty hires who are non-PEGs (purple) and PEGs (orange)according to the criteria used in the IPEDS database.
(TIF)

S1 Table WMPwD and IPED data descriptions. Specifications/categories are followed by the years for which they apply in parentheses. If no year listed, information applies to all years included in analysis.
(PDF)

S2 Table **Contingency tables for search committees with variable demographics:** a) Search committees containing at least one PEER versus hiring at least one PEER; b) Search committee containing at least one PEG versus hiring at least one PEER; c) Search committee containing at least one PEER versus hiring at least one PEG; and d) Search committee containing at least one PEG versus hiring at least one PEG.
(PDF)

S3 Table Longitudinal ratios of PEER students to faculty over institutions. (PDF)

S4 Table T-test values comparing longitudinal ratios of PEER students to faculty. (PDF)

S5 Table Departmental hires before, during, and after CHP, using IPEDS criteria for PEER. (PDF)
