# Supplementary figures and images for: Improving biology faculty diversity through a co-hiring policy and faculty agents of change
Source: PLoS One. 2023 May 15;18(5):e0285602. doi: 10.1371/journal.pone.0285602 (PMC10184900; doi:10.1371/journal.pone.0285602)

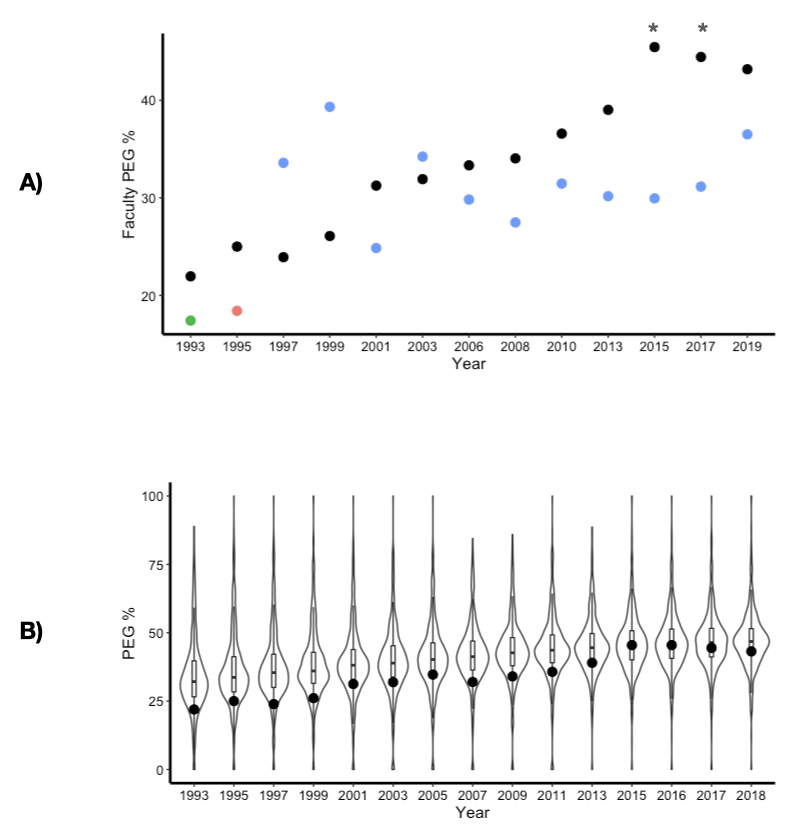

Supplement: S1 Fig — A) The percentages of faculty that are PEGs are shown for both the study department (n = 94 faculty) (black) and nationwide science & engineering doctorate holders (average n = 70,277 faculty per year) (green, red, and blue circles) over time. For the nationwide data, colors represent the survey sample specification–science & engineering doctorate holders employed in all postsecondary institutions (green); science & engineering doctorate holders employed at 4-year colleges and universities (red); and tenured and tenure-track doctorate holders in the biological sciences employed at 4-year colleges and universities (blue). Years with significantly higher PEG percentages in the study department faculty compared to nationwide faculty are indicated by stars. B) The percentage of PEG faculty at the study department (black points) and nationwide faculty at 4-year institutions (violin plots) as reported in IPEDS (average n = 513 institutions per year). (TIF) [file pone.0285602.s001.tif]

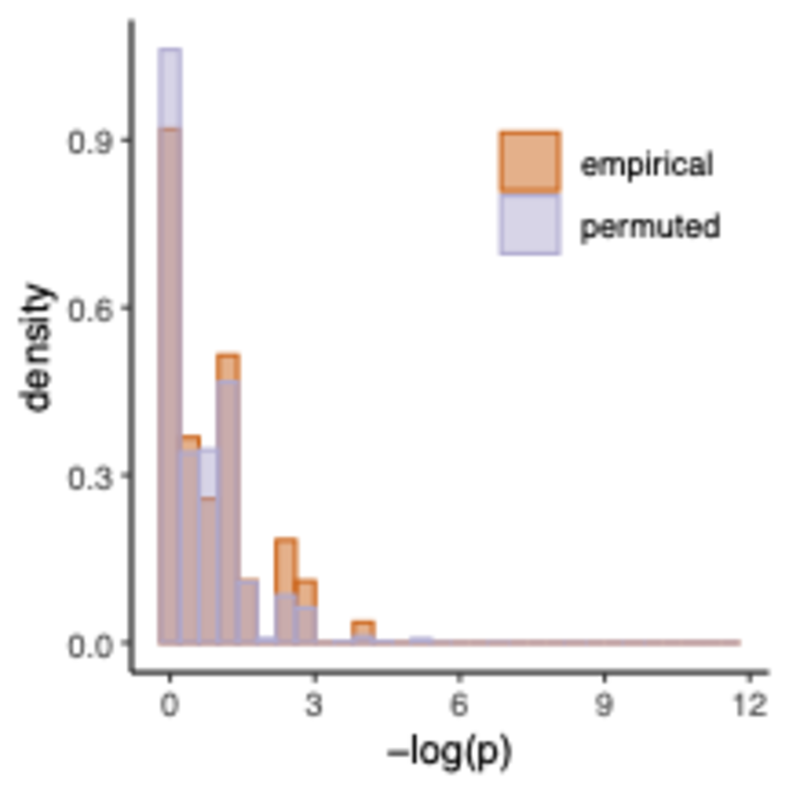

Supplement: S2 Fig — Histogram of AoC -log(p-values). This histogram shows -log(p) from one-tailed Fisher exact tests querying individual search committee membership (n = 68 search committee members) association with hiring at least one PEER (IPEDS criteria) for the empirical historical data (orange) and for the permuted data (10,000 permutations) (purple). (TIF) [file pone.0285602.s002.tif]

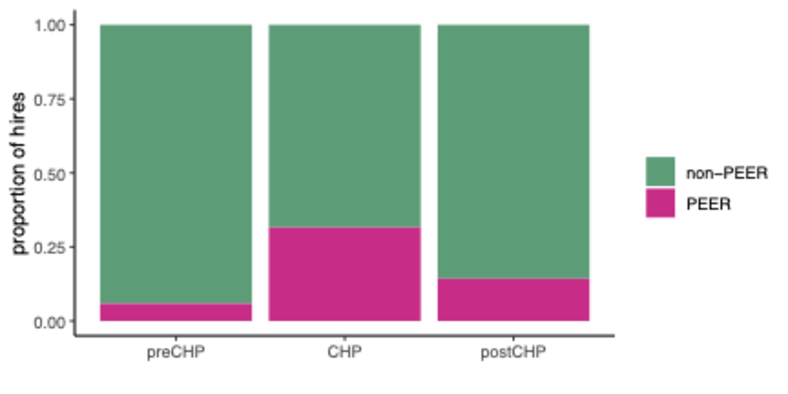

Supplement: S3 Fig — These plots show changes in proportion of faculty hired (n = 69) before the CHP, during the CHP, and after the CHP in terms of a) faculty hires who are non-PEER (green) and PEER (pink) and b) faculty hires who are non-PEGs (purple) and PEGs (orange)according to the criteria used in the IPEDS database. (TIF) [file pone.0285602.s003.tif]
