# Supplementary material for: Improving biology faculty diversity through a co-hiring policy and faculty agents of change
Source: PLoS One. 2023 May 15;18(5):e0285602. doi: 10.1371/journal.pone.0285602 (PMC10184900; doi:10.1371/journal.pone.0285602)
Supplement: S1 Table — Specifications/categories are followed by the years for which they apply in parentheses. If no year listed, information applies to all years included in analysis. (PDF) [file pone.0285602.s004.pdf]

	IPEDS	WMPwD
**Source**	The National Center for Education Statistics Integrated Postsecondary Education Data System	The National Science Foundation National Center for Education Statistics, "Women, Minorities, and Persons with Disabilities Report" from 1996-2021. The data sources for the specific tables are as follows:<br><br>U.S. Department of Education/NCES, National Study of Postsecondary Faculty (1993).<br><br>National Science Foundation, Division of Science Resources Statistics, Scientists and Engineers and Statistical Data System (SESTAT) (1995, 1997, 1999, 2003).<br><br>National Science Foundation, National Center for Science and Engineering Statistics, Survey of Doctorate Recipients (2001, 2006, 2008 [preliminary data], 2010, 2013, 2015, 2017, 2019).

**Population**	Full-time tenured/tenure-track instructional/research/public service faculty employed at institutions with Carnegie Classification of master's Colleges and Universities as of the 2019-2020 school year	Science & Engineering doctorate holders in the biological sciences employed in universities and 4-year colleges:<br><br>Tenured/Tenure Track science & engineering faculty employed – faculty from all disciplines; no university specification (1993)<br><br>Science & engineering faculty (assistant, associate and tenured professors) employed at 4-year colleges and universities (1995)<br><br>Scientists & engineers employed in life and related scientists at 4-year college or universities (1997, 1999, 2001)<br><br>Tenured/Tenure-Track biological/life scientists employed in universities and 4-year colleges (2003, 2006, 2008, 2010, 2013, 2015, 2017)<br><br>Tenured/Tenure-Track biological/agricultural/other life scientists employed in universities and 4-year colleges (2019)

**Sample sizes**	Total number of institutions included per year for PEER analysis:<br>1993: 512, 1995: 514,<br>1997: 512, 1999: 515,<br>2001: 514, 2003: 519,<br>2005: 509, 2007: 511,<br>2009: 511, 2011: 514,<br>2013: 508, 2015: 510,<br>2016: 511, 2017: 512,<br>2018: 510<br><br>Total number of institutions included per year for PEG analysis:<br>1993: 513, 1995: 515,<br>1997: 514, 1999: 518,<br>2001: 517, 2003: 521,<br>2005: 510, 2007: 511,<br>2009: 512, 2011: 514,<br>2013: 508, 2015: 510,<br>2016: 511, 2017: 512,<br>2018: 510	Total number of survey respondents per year:<br>1993: 174000; 1995: 135200;<br>1997: 52400; 1999: 152300;<br>2001: 45750; 2003: 74500;<br>2006: 44600; 2008: 35300;<br>2010: 44500; 2013: 36800;<br>2015: 34900; 2017: 34350;<br>2019: 48350

**Race/Ethnicity categories included in Persons historically Excluded do to Ethnicity and Race (PEERs)**	1993 - 2009: American Indian/Alaska Native, Black non-Hispanic, Hispanic<br><br>2011 - 2018: American Indian or Alaska Native, Black, Hispanic or Latino, Native Hawaiian or Other Pacific Islander	Black (1993-2019), Hispanic or Latino (1993-2019), American Indian or Alaska Native – when data was available (1995-2019), Native Hawaiian or Other Pacific Islander – when data was available (2008-2019)<br><br>Some/all suppressed to avoid disclosure of confidential information: American Indian or Alaskan Native (2006, 2008, 2010, 2013, 2015, 2017, 2019); Native Hawaiian/Pacific Islander (2008, 2010, 2013, 2015, 2017, 2019)<br><br>Suppressed because fewer than 50 weighted cases: American Indian/Alaskan Native (2001, 2003)
**Departments Included**	All departments	Science & Engineering (1993-1995); Biology/life related sciences (1997-2019)
**Institutions**	Institutions with Carnegie Classification of Master's-Granting Colleges and Universities as of the 2019-2020 school year	All postsecondary institutions (1993); 4-year colleges and universities (1995-2019)
