# Supplementary material for: Improving biology faculty diversity through a co-hiring policy and faculty agents of change
Source: PLoS One. 2023 May 15;18(5):e0285602. doi: 10.1371/journal.pone.0285602 (PMC10184900; doi:10.1371/journal.pone.0285602)
Supplement: S2 Table — Contingency tables for search committees with variable demographics: a) Search committees containing at least one PEER versus hiring at least one PEER; b) Search committee containing at least one PEG versus hiring at least one PEER; c) Search committee containing at least one PEER versus hiring at least one PEG; and d) Search committee containing at least one PEG versus hiring at least one PEG. (PDF) [file pone.0285602.s005.pdf]

a) **Search committees containing at least one PEER versus hiring at least one PEER.**

	**non-PEER committee**	**>=1 PEER on committee**
**non-PEER hire(s)**	16	15
**>=1 PEER hire**	4	13

b) **Search committee containing at least one PEG versus hiring at least one PEER.**

	**Committee with no PEG**	**>=1 PEG on committee**
**non-PEER hire(s)**	7	24
**>=1 PEER hire**	2	15

c) **Search committee containing at least one PEER versus hiring at least one PEG.**

	**non-PEER committee**	**>=1 PEER on committee**
**no PEG hire(s)**	15	14
**>=1 PEG hire**	5	14

d) **Search committee containing at least one PEG versus hiring at least one PEG.**

	Committee with no PEG	>=1 PEG on committee
Non-PEG hire(s)	5	24
>= 1 PEG hire(s)	4	15
