# Supplementary material for: Improving biology faculty diversity through a co-hiring policy and faculty agents of change
Source: PLoS One. 2023 May 15;18(5):e0285602. doi: 10.1371/journal.pone.0285602 (PMC10184900; doi:10.1371/journal.pone.0285602)
Supplement: S3 Table — (PDF) [file pone.0285602.s006.pdf]

Year	Study Department Ratio	Median Ratio Among National Institutions	Institutions with Ratio < 1	Total Number of Institutions	Percentage of Institutions Under-Representing PEER Faculty
2003	0.8391132	0.4397202	438	521	84.06909789
2005	0.8082249	0.4454443	442	510	86.66666667
2007	0.8474972	0.4467394	452	511	88.45401174
2009	0.9184174	0.4269388	465	512	90.8203125
2011	0.6825907	0.419596	474	514	92.21789883
2013	0.6389926	0.4009121	476	508	93.7007874
2015	0.5657643	0.3747229	481	510	94.31372549
2017	0.4339409	0.3770396	492	512	96.09375
2018	0.457245	0.3732902	489	510	95.88235294
