# Supplementary material for: Improving biology faculty diversity through a co-hiring policy and faculty agents of change
Source: PLoS One. 2023 May 15;18(5):e0285602. doi: 10.1371/journal.pone.0285602 (PMC10184900; doi:10.1371/journal.pone.0285602)
Supplement: S4 Table — (PDF) [file pone.0285602.s007.pdf]

Years Compared	P-value
2003 to 2005	p-value = 0.8564
2005 to 2007	p-value = 0.7589
2007 to 2009	p-value = 0.1735
2009 to 2011	p-value = 0.5102
2011 to 2013	p-value = 0.1561
2013 to 2015	p-value = 0.5271
2015 to 2017	p-value = 0.5681
2017 to 2018	p-value = 0.9354
2003 to 2018	p-value = 3.384e-06
