# Supplementary material for: Improving biology faculty diversity through a co-hiring policy and faculty agents of change
Source: PLoS One. 2023 May 15;18(5):e0285602. doi: 10.1371/journal.pone.0285602 (PMC10184900; doi:10.1371/journal.pone.0285602)
Supplement: S5 Table — (PDF) [file pone.0285602.s008.pdf]

	Pre-co-hiring period	Co-hiring period	Post-co-hiring period
**PEER hires**	1	12	2
**Total hires**	6	38	14
